# Structural environment built by AKAP12+ colon mesenchymal cells drives M2 macrophages during inflammation recovery

**DOI:** 10.1038/srep42723

**Published:** 2017-02-16

**Authors:** Jun-Mo Yang, Hye Shin Lee, Ji Hae Seo, Ji-Hyeon Park, Irwin H. Gelman, Eng H. Lo, Kyu-Won Kim

**Affiliations:** 1 SNU-Harvard NeuroVascular Protection Research Center, College of Pharmacy and Research Institute of Pharmaceutical Sciences, Seoul National University, Seoul, 151-742, Korea; 2 Department of Cancer Genetics, Roswell Park Cancer Institute, Buffalo, NY 14263, USA; 3 Neuroprotection Research Laboratory, Departments of Radiology and Neurology, Massachusetts General Hospital and Harvard Medical School, Charlestown, MA 02129, USA; 4 Crop Biotechnology Institute, GreenBio Science and Technology, Seoul National University, Pyeongchang 25354, Republic of Korea

## Abstract

Macrophages exhibit phenotypic plasticity, as they have the ability to switch their functional phenotypes during inflammation and recovery. Simultaneously, the mechanical environment actively changes. However, how these dynamic alterations affect the macrophage phenotype is unknown. Here, we observed that the extracellular matrix (ECM) constructed by AKAP12+ colon mesenchymal cells (CMCs) generated M2 macrophages by regulating their shape during recovery. Notably, rounded macrophages were present in the linear and loose ECM of inflamed colons and polarized to the M1 phenotype. In contrast, ramified macrophages emerged in the contracted ECM of recovering colons and mainly expressed M2 macrophage markers. These contracted structures were not observed in the inflamed colons of AKAP12 knockout (KO) mice. Consequently, the proportion of M2 macrophages in inflamed colons was lower in AKAP12 KO mice than in WT mice. In addition, clinical symptoms and histological damage were more severe in AKAP12 KO mice than in WT mice. In experimentally remodeled collagen gels, WT CMCs drove the formation of a more compacted structure than AKAP12 KO CMCs, which promoted the polarization of macrophages toward an M2 phenotype. These results demonstrated that tissue contraction during recovery provides macrophages with the physical cues that drive M2 polarization.

Macrophages are diverse and multifunctional innate immune cells that play a role in many biological processes^1^. While performing their various roles, macrophages can adopt many functional phenotypes. Although the phenotype of macrophages appears as a spectrum^2^, they are generally categorized by activation state as either classically activated (M1, pro-inflammatory) or alternatively activated (M2, anti-inflammatory, tissue healing)^3^. The versatility of macrophages is attributable to their ability to change their functional phenotype in response to the prevailing microenvironment, which is composed of soluble and physical cues. Soluble cues that regulate macrophage phenotype are relatively well determined^4^. For instance, tumor necrosis factor-α (TNF-α), interferon-γ (IFN-γ) and lipopolysaccharide (LPS) drive macrophages to an M1 phenotype, whereas, interleukin- 4 (IL-4) is a cytokine that stimulates M2 polarization. In contrast, it is unclear whether physical environments, such as extracellular matrix (ECM) stiffness, architecture and composition, can affect macrophage polarization^5^.

Inflammation and the recovery process are accompanied by dynamic alterations in the functional phenotype of macrophages and the mechanical environment. During inflammation, activated macrophages derived from circulating monocytes or resident tissue macrophages migrate to damaged tissue and become polarized to an M1 phenotype. After inflammation, the macrophage functional phenotype gradually shifts to an M2 phenotype during the recovery of the tissue^6^. Simultaneously, the mechanical environment is actively changing. The ECM in the vicinity of the damaged tissue is degraded by several proteolytic enzymes and becomes semi-liquid during the inflammation phase^7^. During the recovery phase, this soft ECM become stiff and tight by regeneration and contraction of the tissue^8^. Although these two dynamic changes interacts with each other, the only well-established one-way communications are the promotion of ECM destruction by M1 macrophages and the contribution of M2 macrophages to tissue remodelling by deposition of collagen. Nevertheless, how alteration of the mechanical environment affects macrophages polarization during inflammation and recovery is still largely undetermined.

It was recently shown that ECM structure affects the migratory function of macrophages. For example, during atherosclerosis, M2 populations dominate within the collagen-rich fibrous cap and adventitia surrounding the plaque and exhibit an elongated morphology^9^,^10^. Despite the evidence suggesting that ECM properties are related to macrophage function and phenotype, the mechanism whereby the ECM regulates the functional phenotype of macrophages during specific biological processes has not been explored.

A-kinase anchoring protein 12 (AKAP12) is a scaffold protein that is involved in mechanical processes, such as cytoskeleton remodeling, integrin clustering and the formation of mature focal adhesion^11^,^12^. Its biological functions have also been studied in several disease models. For example, it has been reported to function as a tumor suppressor and our recent study has revealed its protective role in brain injury^13^,^14^. During brain injury, AKAP12 showed higher expression in the recovery phase than in the acute inflammation phase. Moreover, the lesion area of AKAP12 KO mice was less compacted than that of WT mice during the recovery phase following CNS injury. Therefore, we postulated that AKAP12 functions as a regulator of tissue remodeling and contraction, and the mechanical environment regulated by AKAP12 could modulate disease progression by regulating macrophage phenotypes during recovery.

In the present study, we demonstrated the effect of AKAP12+ colon mesenchymal cells on macrophage phenotypes during dextran sodium sulfate (DSS)-induced colitis. Contraction of the matrix stimulated by AKAP12 might be attributable to its ability to regulate the mechanical processes of cells, which is evidenced by the observation that AKAP12 KO mice have a loose ECM structure during intestinal inflammation recovery. We also showed that AKAP12 KO mice were more sensitive to DSS-induced colitis than WT mice, as tissue contraction leads to the generation of M2 macrophages and reduction of the inflammation response during recovery.

## Results

### AKAP12 is highly expressed in colon mesenchymal cells and regulates the ECM

**Figure 1:**
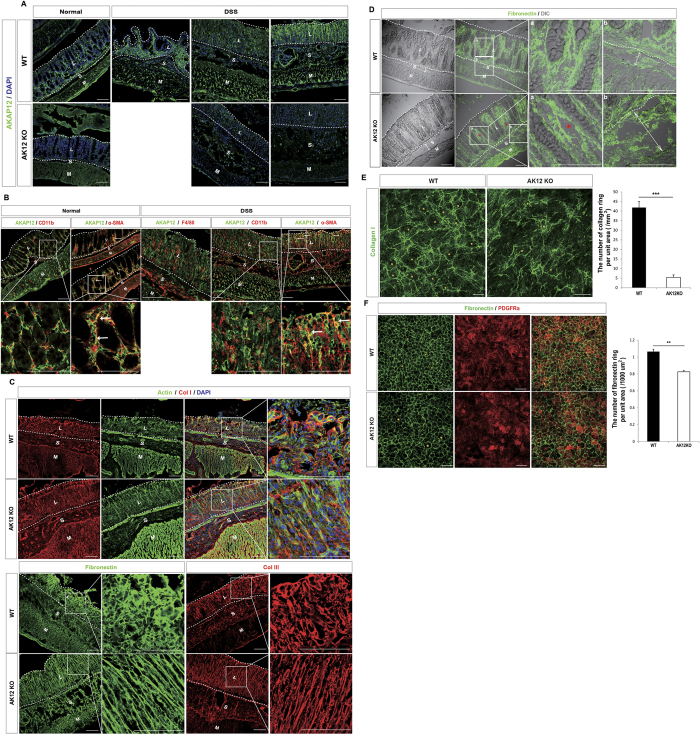
AKAP12+ CMCs regulates ECM organization. (**A**) Confocal imaging of AKAP12 expression in WT and AKAP12 KO colon during normal and inflammation condition. Bars: 100 μm. (**B**) Localizations of F4/80, CD11b, α-SMA with AKAP12 under conditions of normal and inflammation were tested to identify the cell type of AKAP12 (+) cells by confocal microscopy in intestinal inflammation. Co-localizations of α-SMA and AKAP12 were shown by white arrows. Bars: 100 μm. (**C**) Immunostaining for actin, collagen I, fibronectin, collagen III and cell nucleus in WT and AKAP12KO mice mucosa. Bars: 100 μm. ECM in dotted line was different in WT and AKAP12KO mice. (**D**) DIC image with immunostained fibronectin in WT and AKAP12 KO normal colon. Width of submucosa is marked by bi-directional white arrows. Fibronectin aberration is shown by red asterisk. Bars: 100 μm. (**E**) Immunostaining for collagen I in whole colon cell primary culture of WT and AKAPKO mice. Collagen ring structure is indicated by yellow asterisk. (n = 3 for WT or AKAP12 KO mice, respectively) Bars: 300 μm (**F**) Immunostaining for fibronectin and PDGFRα in whole colon cell primary culture of WT and AKAP12 KO mice. Fibronectin ring structure is shown by different sized yellow asterisk between WT and AKAP12 KO culture. (n = 3 for WT or AKAP12 KO mice, respectively) Bars: 100 μm. All images are representative of at least 3 mice per group. P^##^ < 10^−6^, P^#^ < 10^−4^, P*** < 10^−3,^ P** < 10^−2^. Error bars, s.e.m.

We first found that AKAP12 was expressed in both normal and inflamed mouse colons which were injured by ingestion of dextran sodium sulfate (DSS) (Fig. 1A)^15^. The expression of AKAP12 was not observed in myeloid cells but in α-smooth muscle actin (α-SMA) positive mesenchymal cells (Fig. 1B)^16^. Because these cells function as organizers of the tissue matrix^17^, it prompted us to compare the ECM structure in the colon environment of WT and AKAP12 KO mice. Indeed, the collagen and fibronectin structures differed in the mucosa of inflamed colons of WT and AKAP12 KO littermates (Fig. 1C); shortened and tightened ECM structures were observed in WT mucosa, whereas linear and longer structures were observed in AKAP12 KO mucosa. Even in the absence of inflammatory insult, the mucosa of AKAP12 KO colons even showed small aberrations in fibronectin structure. Additionally, the submucosa of AKAP12 KO colon was slightly longer than that of WT colon (Fig. 1D). Moreover, in whole colon primary cultures, the collagen structure derived from AKAP12 KO mice was not connected (Fig. 1E), and the fibronectin structure in the AKAP12 KO culture was larger than that in the WT culture (Fig. 1F). These results imply that AKAP12 (+) CMCs influence ECM organization.

### Tightness of the ECM that is regulated by AKAP12+ mesenchymal cells determines the shape of macrophages

**Figure 2:**
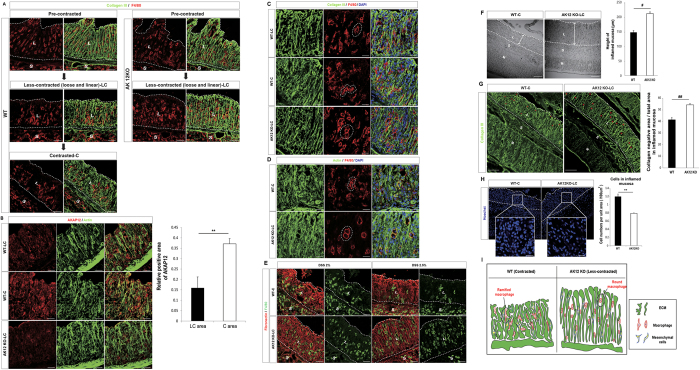
AKAP12+ CMCs mediated tissue contraction promotes ramified macrophages. (**A**) Immunostaining for Collagen III and F4/80 in inflamed colon mucosa of WT and AKAP12KO mice. Pre-contracted, less-contracted and contracted areas were selected according to state of collagen structure. Bars: 50 μm. (**B**) Immunostaining for AKAP12 and actin in WT and AKAP12 KO inflamed colon. Bars: 25 μm. Relative AKAP12 positive area of contracted or less contracted mucosa in WT mice was measured by Image J. (n = 4 for contracted and n = 3 for less contracted mucosa). (**C**) Immunostaining for collagen III, F4/80 and cell nucleus showed that ramified macrophages were in contracted mucosa and rounded macrophages in less-contracted mucosa. Representative macrophages were indicated by dotted line along with its shapes. Bars: 25 μm. (**D**) Immunostaining for F4/80 and actin in WT and AKAP12 KO inflamed colon. Representative macrophages were indicated by dotted line along with its shapes. Bars: 25 μm. (**E**) Immunostaining for fibronectin and F4/80 in WT and AKAP12 KO inflamed colon. Bars: 50 μm. (**F**) Average vertical height of inflamed mucosa were measured (n = 9 or 7 for WT or AKAP12KO, respectively) (mean of 3 different sections used). Bars: 100 μm. (**G**) Proportion of collagen negative area to total area in mucosa was measured. Hollow pores in WT and AKAP12KO collagen structures were marked by red asterisk of different size (n = 6 per group) (mean of 2 technical replicates used). (**H**) Density of cells in mucosa were measured (n = 7 or 5 for WT or AKAP12KO, respectively) (mean of 2 different sections used). Bars: 20 μm. (**I**) Schematic images depicting shapes difference of macrophages between WT and AKAP12 KO inflamed colon mucosa. All imaging experiments were performed by confocal microscopy. L: lamina propria (mucosa), S: submucosa, M: muscularis externa. All panels, WT and AKAP12KO mice were given 2% DSS in their drinking water for 8d and were then allowed to recover with normal drinking water for 4d. All images are representative of at least 5 mice per group. P^##^ < 10^−6^, P^#^ < 10^−4^, P** < 10^−2^. Error bars, s.e.m.

Because inflammation in the DSS-induced colitis model occurs continuously at colon mucosa, the tissue states are heterogeneous. Therefore, several states of recovering colon mucosa could be seen concurrently. Based on the grade of ECM contraction, we subdivided the structures of inflamed colon mucosa into ‘pre-contracted’, ‘less-contracted’ and ‘contracted’ structures (Fig. 2A). The expression of AKAP12 was higher in contracted areas than in less-contracted areas (Fig. 2B). The shape of macrophages was altered between these different structures, implying that mechanical environment may affect macrophage shape (Fig. 2A)^5^. Interestingly, contracted structures with ramified macrophages were only observed in WT colons, while pre-contracted and less-contracted structures with rounded macrophages were observed in both WT and AKAP12 KO colons (Fig. 2C–E). Then, we analyzed the properties of contracted and less-contracted areas. Less-contracted mucosa in AKAP12 KO showed longer height, higher proportion of collagen negative area and lower cell density than contracted mucosa in WT (Fig. 2F–H), which could be the result of deficient contraction of inflamed colons of AKAP12 KO mice. These data indicate that AKAP12-mediated contraction of ECM structures promotes the formation of ramified macrophages rather than rounded macrophages (Fig. 2I).

### Tissue contraction by AKAP12+ mesenchymal cells drives the ramified M2 macrophage

**Figure 3:**
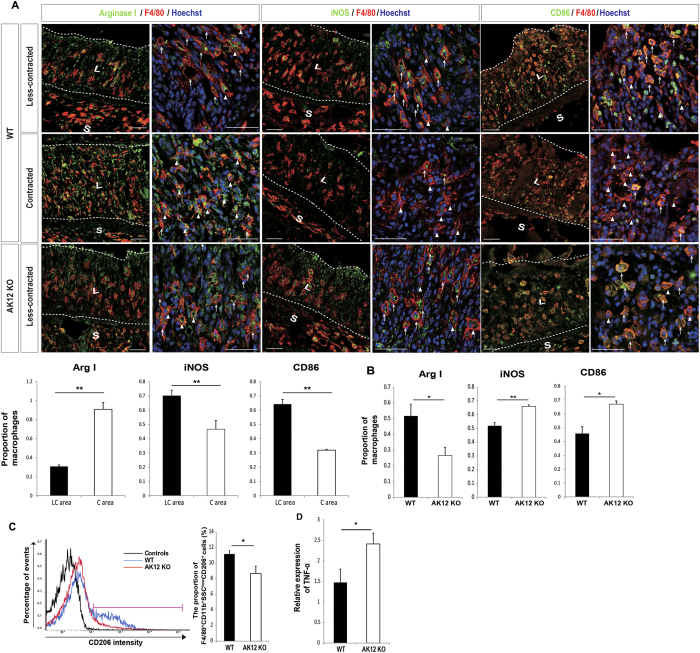
Contracted inflamed colon mucosa generates ramified M2 macrophages. (**A**) Immunostaining for anti-inflammatory (Arginase I) and inflammatory markers (iNOS and CD86) of macrophages in inflamed colon mucosa of WT and AKAP12 KO mice. Less-contracted and contracted mucosa was selected according to macrophage shapes. Arg1+ macrophages and Arg1− macrophages are marked by arrowheads and arrows, respectively. iNOS+ or CD86+ macrophages and iNOS− or CD86− macrophages are marked by arrows and arrowheads, respectively. Bars: 50 μm. Proportions of arginase I, iNOS and CD86 positive macrophages to total macrophages in less-contracted and contracted mucosa of WT mice were calculated (n = 3 per all group). (**B**) Proportions of arginase I+, iNOS+ and CD86+ macrophages to total macrophages in WT and AKAP12 KO inflamed mucosa were calculated (n = 5 for CD86+ and n = 4 for iNOS+ in AKAP12 KO inflamed mucosa, n = 3 per all rest group). (**C**) Representative FACS plot for CD206 positive macrophages in WT and AKAP12 KO inflamed colon. Macrophages within pink bars were considered as M2 macrophages. CD206-negative controls are shown in black (n = 4 per group). (**D**) TNFα mRNA expression in WT and AKAP12 KO inflamed colon were detected by Real-time PCR (n = 4 per group). L: lamina propria (mucosa), S: submucosa. All images are representative of at least 5 mice per group. All panels, P** < 0.01, P* < 0.05. Error bars, s.e.m.

Alterations in cell shape have long been associated with changes in cell function^18^. In many studies, ramified or elongated macrophages are reported to polarize to M2 phenotypes. Therefore, to determine the relationship between macrophage shapes and functional phenotypes^19^, we examined several macrophage markers in each mechanical environment. Interestingly, the proportion of arginase I+ macrophages (M2 macrophages) was higher in contracted areas with ramified macrophages than in less-contracted areas with rounded macrophages. In contrast, the population of inflammatory macrophages marked by iNOS and CD86 was higher in less-contracted areas than in contracted areas (Fig. 3A). Therefore, the population of arginase I+ M2 macrophages was lower and that of iNOS+ and CD86+ M1 macrophages was higher in AKAP12 KO inflamed mucosa than in WT (Fig. 3B). The proportion of CD206+ M2 macrophages among the total macrophages was also higher in WT inflamed colons than in AKAP12 KO (Fig. 3C, Supplementary Fig. 1). In addition, the relative expression of TNF-α, a pro-inflammatory cytokine, was higher in AKAP12 KO inflamed colon (Fig. 3D). Collectively, these data show that contracted tissue drives an anti-inflammatory environment by generating ramified M2 macrophages.

### AKAP12 KO mice have increased sensitivity to DSS-induced colitis

**Figure 4:**
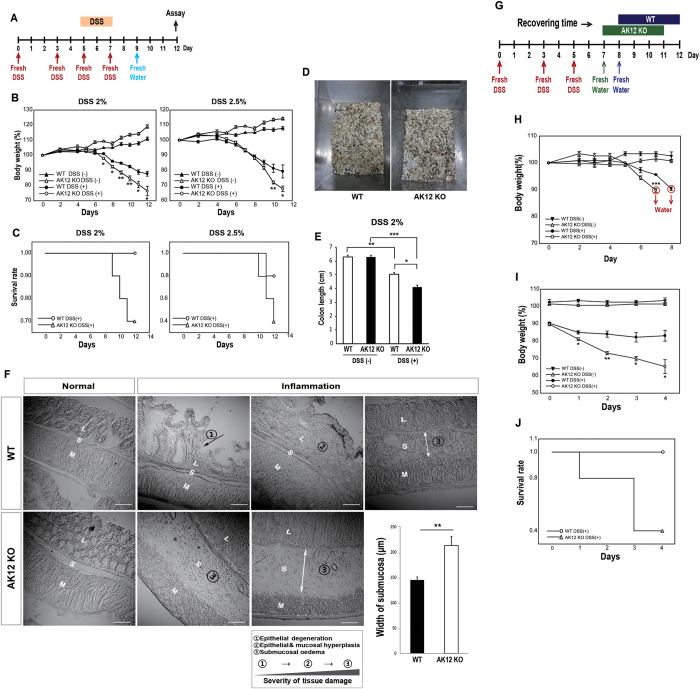
Protective role of AKAP12 during intestinal inflammation recovery. (**A**–**F**) WT and AKAP12 KO mice were given 2 or 2.5% DSS in their drinking water with control group (normal water) for 8 days and were then allowed to recover with normal drinking water for a further 4 days (n = 10 per group for DSS 2%, n = 5 per group for DSS 2.5% and n = 5 per group for controls). (**A**) Schedule of DSS ingestion to WT and AKAP12 KO mice. (**B**) Percentage of body weigh changes in WT and AKAP12 KO mice after given 2 or 2.5% DSS in water. (**C**) Proportion of survived mouse to total mouse in each groups were recorded at each days. (**D**) Picture of blood stools in cage of DSS induced WT and AKAP12 KO mice were taken. (**E**) Colon lengths were recorded by using ruler on day 12 (n = 5 or 4 for WT or AKAP12KO, respectively). (**F**) Tissue states were marked in images and numbered with order of tissue damage. Width of submucosa in image was measured by ruler and then the value was converted to real value by using scale (n = 5 per group). Bars: 100 μm. (**G**–**J**) WT and AKAP12 KO mice were given normal water for further 4 days after given 2% DSS until body weight of each group decreased to 90% compared to initial body weight or after given normal water (n = 5 per group for DSS 2% and n = 5 per group for controls). (**G**) Schedule for comparing the recovering ability of WT and AKAP12 KO mice directly. AKAP12 KO mice were given 2% DSS for 7 days and WT mice for 8 days and then, changed DSS water to normal water. (**H**) Body weights of WT and AKAP12 KO were decreased to 90% at 8 day and 7 day, respectively (red circle). (**I**) Body weight changes of WT and AKAP12 KO mice in recovering period were shown. (**J**) Proportion of survived mouse to total mouse in each groups were recorded at each days. L: lamina propria, S: submucosa, M: muscularis externa. All panels, P* < 0.05, P** < 0.01, P*** < 10^−3^. Error bars, s.e.m.

We next compared the clinical symptoms of colitis in WT and AKAP12 KO mice to determine whether differences in the mechanical environment were correlated with the severity of intestinal inflammation. Mice ingested 2% or 2.5% DSS in water for 8 days, followed by fresh water for 3 days (Fig. 4A). There was no obvious difference in water consumption (Supplementary Fig. 2A). The clinical symptoms were more severe in AKAP12 KO mice, mirrored by greater weight loss, lower survival, and more blood in stool (Fig. 4B–D). In addition, repetitive DSS-induced colitis experiments confirmed that AKAP12 KO mice were more sensitive to colitis in most experiments (Supplementary Table). Colon length, which indicates the degree of colon damage, was also shorter in colitis-induced AKAP12 KO mice than in WT mice, whereas it was similar in both genotypes under normal conditions (Fig. 4E, Supplementary Fig. 2B). Epithelial degeneration and mucosal hyperplasia were only observed in WT inflamed mucosa, and the average width of the submucosa (an indicator of the degree of submucosal edema) was longer in AKAP12 KO inflamed colon than in WT, implying more severe histological damage in AKAP12 KO inflamed colon than in WT (Fig. 4F).

### Protective role of AKAP12 contributes to recovery of inflamed colon

Interestingly, the difference in the body weight changes between WT and AKAP12 KO mice gradually increased in the later phase of colitis, during which tissue healing and contraction typically occur in a large portion of the inflamed colon. To identify whether the severe damage in AKAP12 KO mice is caused by reduced recovery, we changed the DSS water to fresh water for each mouse when it reached 90% of its initial body weight (Fig. 4G,H). When the clinical signs were compared after the time point at which fresh water was supplied, WT mice showed a mild decrease in body weight after 1 day, which was maintained thereafter. In contrast, AKAP12 KO mice showed a spontaneous decrease in body weight, which was accompanied by a dramatically reduced survival rate (Fig. 4I,J). These data show that AKAP12 has a protective role in the tissue remodeling phase of DSS-induced colitis.

### AKAP12 (+) CMCs promote collagen gel compaction that generates M2 macrophages

**Figure 5:**
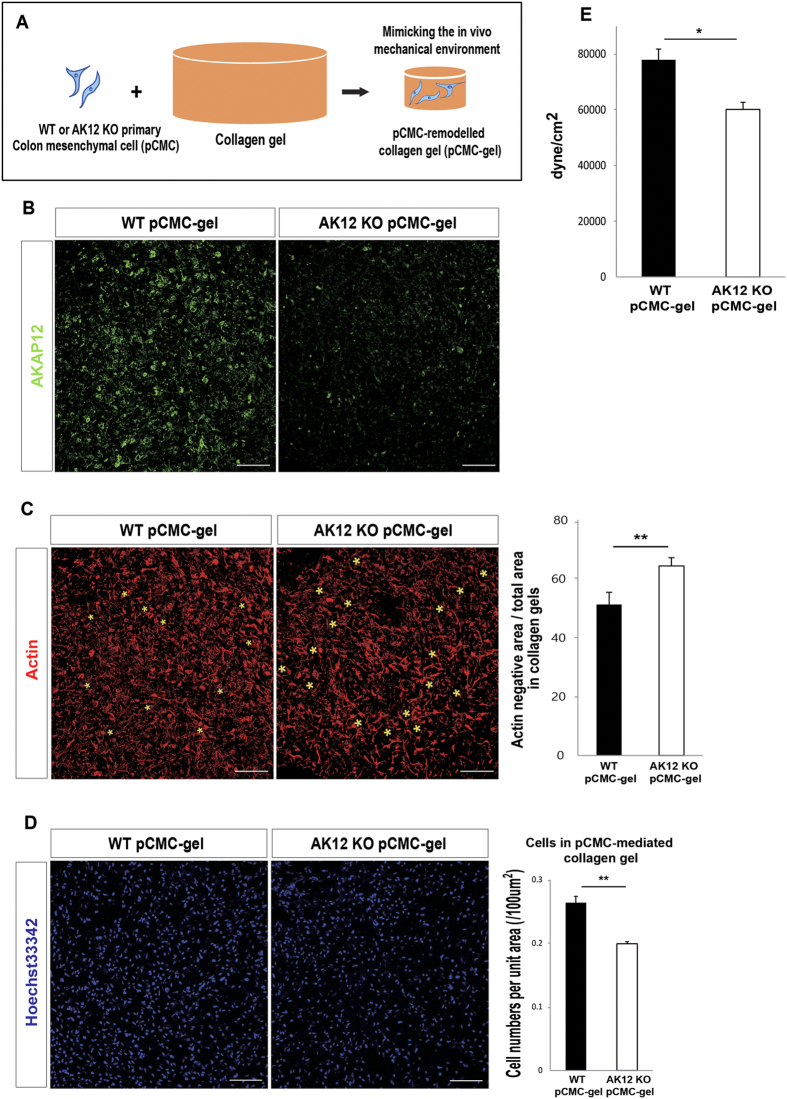
Mechanical difference between WT pCMC-gels and AKAP12 KO pCMC-gels. (**A**) Experimental design of collagen gel assay with pCMCs. To establish pCMC-gel, 7 × 10^5^ WT or AKAP12 KO pCMCs were inserted in 3 mg/ml collagen gels, released from plate after incubated during 8 hr and then further incubated until 48 hr. (**B**) Immunostaining for AKAP12 in WT or AKAP12 KO pCMC-gels. Bars: 100 μm. (**C**) Actin negative area to total area of WT pCMC and AKAP12 KO pCMC-gels with was measured. Actin-negative area in representative images of each gels were marked by yellow asterisk with different size. Bars: 100 μm (**D**) Density of cells in collagen gels per each group were measured by calculate the number of cells per area. Bars: 100 μm (**E**) The storage modulus at 1% strain and at 1 Hz of fixed WT and AKAP12 KO pCMC-gels were averaged. (mean ± SEM, n = 6 per group). All panels, P** < 0.01, P* < 0.05. Error bars, s.e.m.

Next, to demonstrate that the phenomenon is caused by AKAP12+ mesenchymal cells, not by either a difference in the degree of inflammation or the macrophage itself, we designed an *in vitro* experiment to assess macrophage polarity in different mechanical environments. As the remodeling of collagen gel mimics tissue contraction^20^,^21^, it is an appropriate model to verify our *in vivo* results. First, we obtained primary colon-derived mesenchymal cells (pCMCs) from WT and AKAP12 KO colons and allowed the pCMCs to remodel the collagen gel (Fig. 5A). Similar to *in vivo* results, WT pCMC remodeled collagen gels (WT pCMC-gel) highly expressed AKAP12 (Fig. 5B) and showed more dense and compacted structures than AKAP12 KO pCMC remodeled gels (AKAP12 KO pCMC-gel) (Fig. 5C,D). We also observed that collagen gels remodeled by WT pCMCs were actually stiffer than gels remodelled by AKAP12 KO pCMCs (Fig. 5E, Supplementary Fig. 3A,B).

### AKAP12 (+) CMC-mediated-gel compaction drives macrophage M2 polarization and reduces inflammatory response

**Figure 6:**
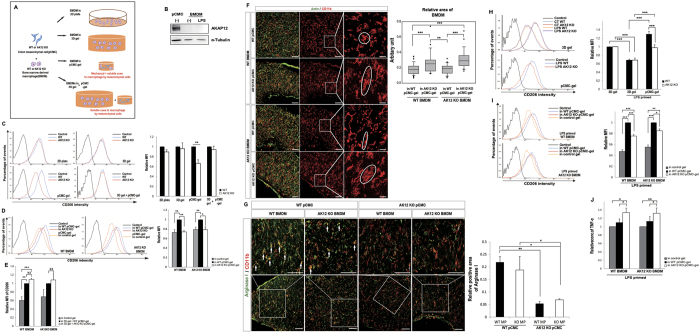
The difference of macrophage polarity between WT pCMC–gels and AKAP12 KO pCMC-gels. (**A**) Experimental design of collagen gel assay with pCMCs and BMDMs. (**B**) Immunoblots of AKAP12 and α-tubulin in lysate of WT pCMCs and BMDMs. (**C**) Relative CD206 intensity of WT (blue) and AKAP12KO (red) BMDMs in 2D plate, 3D gel, pCMC-gels and 3D gel with pCMC-gels. (n = 3, n = 6, n = 7 and n = 3, respectively). (**D**) Relative CD206 intensity of WT and AKAP12 KO BMDMs in control gel (orange), WT pCMC-gel (blue) and AKAP12 KO pCMC-gel (red). (n = 4 per group). (**E**) Relative CD206 intensity of WT or AKAP12 KO BMDMs in gels, WT pCMC-gels or AKAP12 KO pCMC-gels measured by FACS (n = 3 per group). (**F**) Representative confocal imaging of WT or AKAP12 KO BMDMs in WT or AKAP12 KO pCMC-gels. Representative macrophages are magnified. Bars: 100 μm. Box plots of relative area of WT or AKAP12 KO BMDMs in WT or AKAP12 KO pCMC-gels. At least 28 macrophages were selected per group. (**G**) Immunostaining for arginase I and CD11b of WT or AKAP12 KO BMDMs in WT or AKAP12 KO pCMC-gels. Bars: 100 μm. Relative Arginase I positive area of remodeled collagen gels was measured by Image J. (n = 3 per all group). (**H**) Relative CD206 intensity of WT (blue in upper plots) or AKAP12 KO (red in upper plots) BMDMs in gel or LPS primed WT (orange in upper plots) or AKAP12 KO (purple in upper plots) BMDMs in gel and LPS primed WT (blue in lower plots) or AKAP12 KO (red in lower plots) BMDMs in pCMC-gels. (n = 3 per group). (**I**) Relative CD206 intensity of LPS primed WT or AKAP12 KO BMDMs in control gel (orange), WT pCMC-gel (blue) and AKAP12 KO pCMC-gel (red). (n = 3 per group). (**J**) Relative expressions of TNF-α in media of WT or AKAP12 KO BMDMs in control gels, WT pCMC-gels or AKAP12KO pCMC-gels are assessed by ELISA (n = 3 per group). All panels, FACS plots of CD206 are representative. CD206-negative controls are shown in black. CD206 fluorescence intensities of macrophages are relative value. P^#^ < 10^−5^, P*** < 10^−3^, P** < 10^−2^, P* < 0.05. Error bars, s.e.m.

To characterize macrophage polarity in the different mechanical environments, WT or AKAP12 KO bone marrow-derived macrophages (BMDMs) were inserted into two-dimensional (2D) plates, three-dimensional (3D) gels, pCMC-gels and 3D gels with pCMC-gels (Fig. 6A). In accordance with previous results, AKAP12 expression was not detected in BMDMs but was detected in pCMCs (Fig. 6B). Collagen gels were remodeled by pCMCs but not by BMDMs alone, implying that the difference in macrophage polarity between inflamed colons of WT and AKAP12 KO mice was due to AKAP12+ CMCs (Supplementary Fig. 4A). After remodeling of collagen gels for 2 days, we isolated the inserted BMDMs and analyzed their CD206 expression (Supplementary Fig. 4B). The CD206 expression levels of WT and AKAP12 KO BMDMs in 2D plates and 3D gels were similar. There was no significant difference in CD206 expression levels in WT and AKAP12 KO BMDMs inserted into 3D gels and cultured with WT and AKAP12KO pCMC-gels, respectively. Notably, we found a significant difference in the expression of CD206 in the WT and AKAP12 KO BMDMs inserted into WT and AKAP12 KO CMC-gels, respectively (Fig. 6C), implying that the difference of mechanical environment generated by tissue remodeling promotes the difference in macrophage polarity. Then, to verify that this difference in macrophage polarity was caused by pCMCs rather than BMDMs, we measured CD206 expression in MPs of WT or AKAP12 KO pCMC-gels. CD206 expression in both WT and AKAP12 KO BMDMs was lower in AKAP12 KO pCMC-gels than in WT pCMC-gels (Fig. 6D), and this difference between WT and AKAP12 KO BMDMs was not observed when cultured in 3D gels with WT or AKAP12 KO pCMC-gels (Fig. 6E).

We examined the shape of each macrophage in the pCMC-gels. Since *in vitro* differentiated macrophages are already ramified and elongated, we were unable to detect rounded macrophages in collagen gels. Instead, we found that the area of each macrophage in the AKAP12 KO pCMC-gels was larger than those in the WT pCMC- gel, probably due to the loose matrix structure of gels (Fig. 6F). In addition, the levels of arginase I expression were higher in macrophages within WT pCMC-gels than in those within AKAP12 KO pCMC-gels (Fig. 6G). These results show that AKAP12 expression in mesenchymal cells promotes M2 polarization by building a tight mechanical structure in 3D collagen gels.

To confirm these effects in conditions mimicking inflammation and tissue regeneration, we generated lipopolysaccharide (LPS)-primed BMDMs by treating BMDMs with LPS 1 day before mixing with collagen and pCMCs. CD206 expression of both LPS-primed WT and AKAP12 KO BMDMs in collagen gels decreased similarly compared to WT or AKAP12 KO BMDMs in the collagen gels. The reduced CD206 expression of LPS-primed BMDMs was more restored in WT pCMC-gels than in AKAP12 KO pCMC-gels (Fig. 6H). We also tested the CD206 expression of LPS-primed BMDMs in the two pCMC-gels. CD206 expression in BMDMs of WT pCMC-gels was higher than that of AKAP12 KO pCMC-gels, regardless of macrophage genotypes (Fig. 6I). Furthermore, TNF-α secretion from both WT and AKAP12 KO LPS-primed BMDMs was higher in AKAP12 KO pCMC-gels than in WT pCMC-gels (Fig. 6J). Taken together, these results indicate that the physical environment built by AKAP12+ pCMCs in collagen gels establishes a less inflammatory environment by skewing macrophages to an M2 phenotype.

### Bioinformatics suggest that AKAP12 expression is correlated with mechanical-related pathways and M2 marker

**Figure 7:**
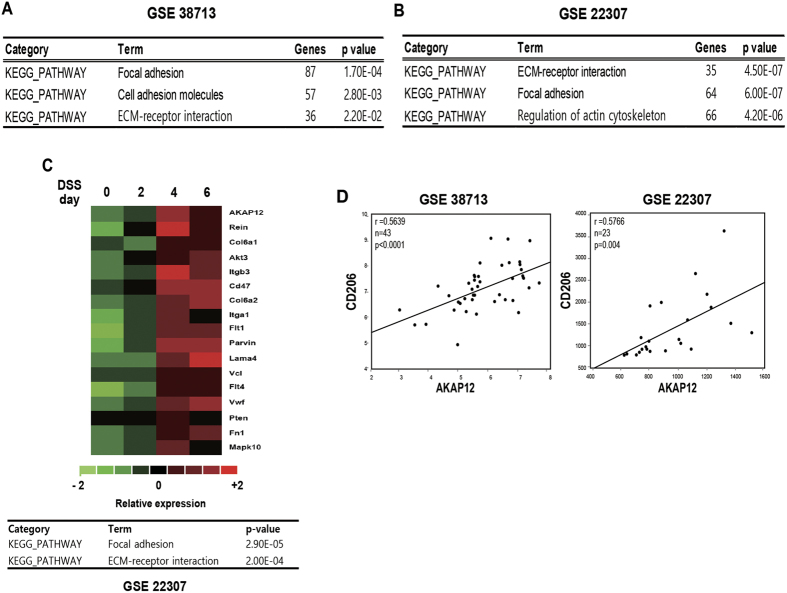
Bioinformatic approach showed the active alteration of mechanical environment in intestinal inflammation and the correlation of AKAP12 with that changes and CD206 expression. (**A**) KEGG pathway shows involvement pathway of differentially expressed genes (DEGs) between non-patients and ulcerative colitis patients (Total genes: 7045, p < 0.001). Analysis of DEGs was performed by GEO2R. (**B**) KEGG pathway shows involvement pathway of differentially expressed gene in DSS 0, 2, 4 and 6 day (Total genes: 3899, p < 0.001). Analysis of DEGs was performed by GEO2R. (**C**) Heatmap of gene expressions which is positive, none and negative correlated with AKAP12 expression in DSS 0, 2, 4 and 6 day of GEO dataset (GSE22307). (**D**) Heatmap of genes highly correlated with AKAP12 expression and involved in focal adhesion and ECM-receptor interaction in DSS induced colitis mice of GEO dataset (GSE22307). KEGG pathway shows involvement pathway of highly correlated genes with AKAP12 expression (Total genes: 418, r > 0.8 with AKAP12). (**E**) Scatter plots showing the correlation of AKAP12 expression with CD206 expression in a GEO dataset (GSE38713, GSE22307). The r value was calculated via Spearman’s rank correlation coefficient analysis.

In addition, bioinformatic analysis of the publicly available GEO database showed that the gene clusters that were significantly changed during intestinal inflammation were involved in focal adhesion, ECM-receptor interaction, and cytoskeletal regulation (Fig. 7A,B). Gene clusters highly correlated with AKAP12 expression in colitis were also involved in focal adhesion and ECM-receptor interaction (Fig. 7C). Furthermore, the expression of CD206, a M2 macrophage marker, was positively correlated with AKAP12 expression in both human and mouse (Fig. 7D).

## Discussion

Although various and complex microenvironmental factors have an influence on macrophage polarization, much of our current understanding is limited to how soluble factors affect macrophage function. Because of this imperfect understanding regarding macrophage polarization, selectively targeting macrophages in disease states has proven unsuccessful thus far^22^. Therefore, to establish targeting of macrophage polarization as a promising therapeutic strategy, it is necessary to consider other factors that modulate the functional phenotypes of macrophages. Many diseases and biological processes, including cancer, atherosclerosis and tissue repair, are accompanied by dynamic changes in the mechanical microenvironment and macrophage phenotypes^3^,^23^,^24^. Recent studies clearly demonstrated that mechanical cues in the microenvironment regulate macrophage phenotype and function. Furthermore, physical cues can also function synergistically with soluble factors to control the macrophage polarization. It has recently been proposed that many mechanical factors such as matrix architecture, substrate stiffness, substrate topography, cell shape and intracellular mechanics control macrophage polarization under *in vitro* conditions^5^. However, the influence of the actively changing physical environment on macrophage functions during biological processes is poorly understood.

**Figure 8:**
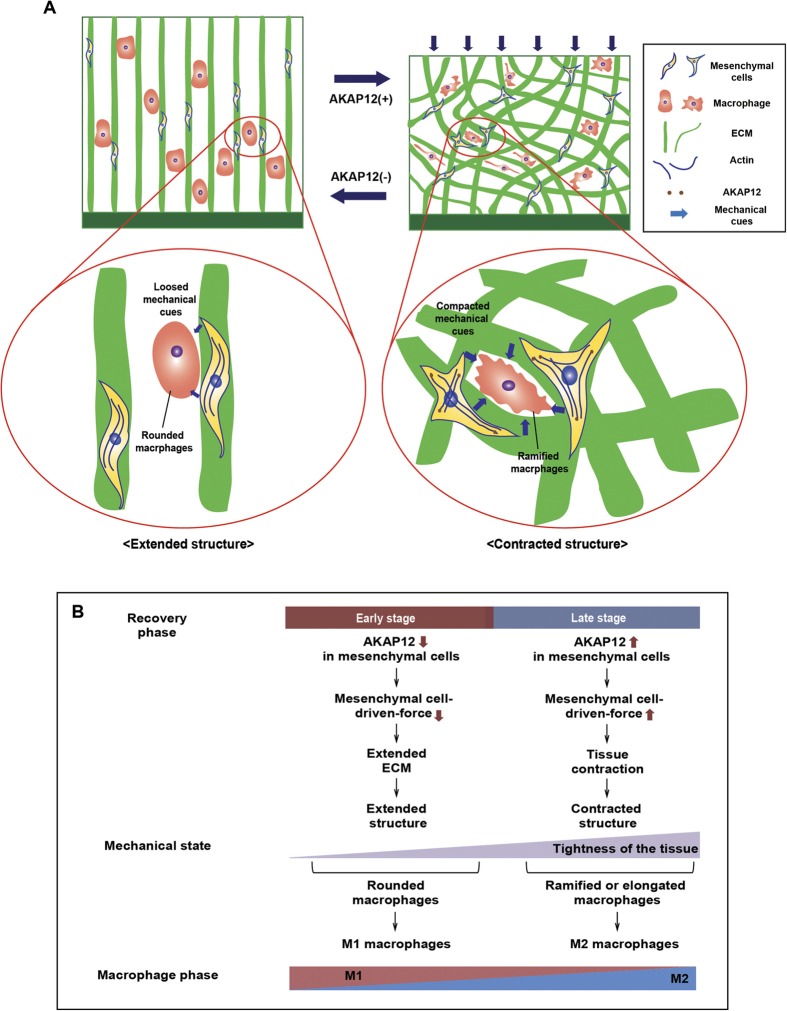
Schematic depiction models that contracted ECM structure built by AKAP12 drives macrophage to less inflammatory phenotype through providing physical cues when tissue healing is ongoing. (**A**) Schematic models depicting effect of AKAP12 on tissue structure that provides macrophages to physical cues. AKAP12 induces the tissue contraction that generates contracted mechanical cues. Then, macrophages sensing these cues shift to their shapes as ramified form and polarize their functional phenotype to M2 side. (**B**) The diagram for the regulation mechanism of mechanical dynamics and macrophage phenotypes during recovery phase. In early stage of recovery, AKAP12 expression was downregulated, which promotes extended ECM structures. These loose structures generate rounded macrophages, skewing to M1 macrophage. On the other hand, in late stage of recovery, the expression of AKAP12 is restored in mesenchymal cells which exert larger forces through larger focal adhesion. These forces generate tissue contraction which contributes to produce ramified M2 macrophage.

In this study, we found that AKAP12 functions as an ECM regulator during tissue contraction. ECM contraction stimulated by AKAP12+ mesenchymal cells subsequently provides physical cues that promote ramified and non-inflammatory macrophages. In contrast, under AKAP12-deficient conditions, the ECM structure has a loose and extended structure, which stimulates the production of rounded macrophages rather than ramified macrophages (Fig. 8A). Thus, AKAP12+ mesenchymal cells contribute to ameliorating intestinal inflammation through generating M2 macrophages indirectly during the late stage of recovery (Fig. 8B). Bioinformatics data also support the hypotheses that the mechanical environment is significantly altered during intestinal inflammation and that AKAP12 expression is linked to M2 macrophage phenotypes in both human and mouse. In addition, as M2 macrophages also promote tissue remodeling, these macrophages and tissue contraction form a positive feedback loop during recovery. Our findings provide a valuable insight for understanding the role of a dynamically changing physical environment on the functional phenotype of macrophage during recovery.

The particular part of inflamed colon tissue displays lower AKAP12 expression than other part of inflamed colon (Fig. 1A) as the AKAP12 expression is higher in contracted mucosa than in less-contracted mucosa (Fig. 2B). After inflammation, cells proliferate to regenerate the tissue and then migrate to appropriate location. At that phase, AKAP12 expression should be decreased because AKAP12 has ability to inhibit the cell migration^25^. That could be the reason why the AKAP12 expression is decreased in less-contracted tissue in which cells still need to migrate to proper location. After positioning, tissue start to contract and AKAP12 seemed to be up-regulated at this tissue remodeling phase, which contributes to this tissue contraction through focal adhesion maturation^12^.

The AKAP12 expression in colon mesenchymal cells is originally high in normal colon. That expression is predicted to be down-regulated when cells need to move and be restored when cells are stabilized. However, in DSS-induced colitis, it is technically difficult to identify the AKAP12 expression in colon mesenchymal cells since the proportion of mesenchymal cells is changing during inflammation and recovery. Therefore, it is ambiguous that alteration of total AKAP12 expression is caused by AKAP12 expression in colon mesenchymal cells and/or changing of its population. In addition, as previous mentioned above, continuous damages by intermittent ingested DSS in water make the heterogeneous tissue state of inflamed colon and the total expression of AKAP12 is sum of these all tissue part. Due to these difficulties, we thought that identifying the change of AKAP12 expression in colon mesenchymal cells during DSS-induced colitis is not a simple process and need to be troubleshot.

Much of our current understanding of the influence of the mechanical environment on macrophage phenotype has come from the field of biomaterials. The shape of macrophages is rounded on a flat substrate but becomes elongated on rough and fibrous substrates^5^. In addition, elongated macrophages show an increase in M2 properties and a reduction in M1 properties^19^. Accordingly, our results indicate that tissue contraction provides a rough and fibrous substrate for macrophages, which in turn generates ramified macrophages (Fig. 2A). These ramified macrophages showed M2 macrophage properties similar to the *in vitro* results (Fig. 3A). However, there is a difference in the shapes of ramified and elongated macrophages. We propose that this difference is attributable to the dimension effect. Elongated macrophages in the 2D condition correspond to ramified macrophages in the 3D condition.

Although mechanical differences were observed between the DSS-induced colitis of WT and AKAP12 KO mice, *in vivo* conditions trigger many other differences between induced colitis in WT and AKAP12KO, which may influence macrophage polarization. Therefore, we designed experimentally remodeled collagen gels using WT or AKAP12 KO CMCs. Collagen gel contraction mimics matrix reorganization during the tissue-healing phase^20^. In addition, among CMCs and BMDMs inserted into collagen gels, only CMCs have the ability to organize collagen gels (Supplementary Fig. 4A), AKAP12 is only expressed in CMCs (Fig. 6B), and WT or AKAP12 KO BMDMs did not display differences in polarity (Fig. 6C). However, there is still the possibility that secretion of a soluble factor from different CMCs affects the functional phenotype of macrophages. To overcome this problem, BMDMs and CMCs were cultured in the same well, but in different collagen gels, which can only share soluble factors. The effect of soluble factors from the CMCs of WT or AKAP12 KO mice on macrophage polarization was similar (Fig. 6E). Thus, through the *in vitro* experiments performed under limited experimental conditions, we confirmed our *in vivo* result that AKAP12+ mesenchymal cells drive M2 macrophage generation through modulating the mechanical environment. In collagen gels, the shape of macrophages in both the WT CMC-gel and AKAP12 KO CMC-gel was ramified, although cell size differed (Fig. 7F). Although the CD206 expression normalized to macrophages in the WT CMC-gel is relatively lower in the AKAP12 KO pCMC-gel than in the WT pCMC-gel, absolute CD206 expression of both macrophages in the WT pCMC-gel and AKAP12 KO pCMC-gel was very high (Fig. 6C). Typically, primary bone marrow-derived macrophages are highly polarized to the M2 phenotype. This is the reason we assumed that macrophages inserted in the WT pCMC-gel polarized to a deeper M2 side than macrophages in the AKAP12 KO pCMC-gel

Further studies need to determine how AKAP12+ mesenchymal cells promote tissue contraction. Mesenchymal cells may need strong forces to contract the matrix as the external environment is converted to a denser matrix during recovery. AKAP12 has been reported to co-localize with focal adhesions and to promote focal adhesion maturation, which generates strong forces in cells^12^,^26^,^27^. We already found that AKAP12 is co-localized with focal adhesions in CMCs (data not shown) and intend to examine the mechanism whereby AKAP12 regulates focal adhesion maturation in CMCs. Moreover, upstream regulators of AKAP12 expression in intestinal inflammation need to be identified. We recently reported that expression of AKAP12 is regulated by retinoic acid (RA) and transforming growth factor-β1 (TGF-β1) in CNS injury. Raldh2, an RA biosynthetic enzyme, was reported to be upregulated in M2 macrophages. Consistent with this previous study, we recently found that ramified macrophages in contracted colon mucosa also expressed more Raldh2 than rounded macrophages in less-contracted mucosa, implying that there are higher RA concentrations in contracted mucosa than in less-contracted mucosa (data not shown). TGF-β1 was previously reported to be expressed in inflamed colons^28^ and to play a role in tissue recovery^29^,^30^. RA and TGF-β1 are known to ameliorate several inflammatory diseases. There is thus a possibility that RA and TGF-β1 regulate the immune response and inflammation via AKAP12. Therefore, elucidating the connections between AKAP12, RA and TGF-β1 will provide insights for uncovering the mechanism of intestinal inflammation.

In this study, we used a DSS-induced colitis model that resembles the pathogenesis of IBD. IBD is a complex disease that occurs as a result of the interaction of environmental and genetic factors leading to immunological responses and inflammation in the intestine^31^. Therefore, IBD may require immunosuppression and a reduction of inflammation for the control of symptoms with anti-inflammatory drugs. Our results demonstrate that the physical environment contributes to regulation of intestinal inflammation. Bioinformatic analysis showed that active mechanical changes and pathways related to mechanical function occur in IBD patients. Thus, regulation of the physical environment may represent a new therapeutic target for intestinal inflammation.

## Materials and Methods

### Mice

Breeding colonies of WT and AKAP12 KO mice (C57BL/6J background) donated by Irwin H. Gelman were established and used for comparison experiments. All mice were maintained in an SPF room in the animal-housing facilities at the Seoul National University. Animal experiments were approved by the Committee for Care and Use of Laboratory Animals at the Seoul National University, according to the Guide for Animal Experiments edited by the Korean Academy for Medical Sciences.

### Induction and assessment of DSS colitis

Mice were given 2% DSS (molecular weight: 36,000-50,000; MP Biomedicals) in their drinking water, and mice were weighed every 24 h. Colitis severity was assessed by several clinical and histological parameters. Body weight changes and survival rate were shown as the mean weight loss to initial body weight and ratio of surviving mice until each day, respectively. Other clinical feature such as decreased movements, rectal bleeding, and anemia were recorded. The length of colon and submucosa was measured after 12 days. Histological features such as epithelial degeneration, epithelial hyperplasia and submucosa edema were compared using a confocal microscope

### Tissue harvesting and histology

Anesthetized mice were perfused with 0.1 M PBS (pH 7.4). Tissue samples of colon were prepared by using the ‘swiss roll’ method as described previously^32^. Isolated colons were cleaned by flushing PBS through the tissue and opened longitudinally. Then, opened colon was rolled on wooden stick and fixed with 4% PFA at 4 °C. After dehydration with serial gradients of sucrose, the colons were embedded in OCT compound (Sakura) and 10-μm-thick colon cryosections made for immunofluorescence staining.

### Immunofluorescence

Colon frozen sections and collagen sections were blocked in staining solution (1% BSA, 0.5% Triton X-100 in PBS) and incubated with primary antibodies against AKAP12 (I. Gelman, Roswell Park Cancer Institute), Fibronectin (DAKO), α-smooth muscle actin (DAKO), Collagen I (Abcam), Collagen III (Abcam), CD11b (AbD Serotec), F4/80 (AbD Serotec), iNOS (BD), Arginase I (Santa cruz), PDGFR-α (R&D systems), CD86 (Biolegend) and phalloidin (Molecular Probes) at RT for 1 h followed by Alexa 488, 546 and 350 (Invitrogen) secondary antibodies at RT for 1 h. Nuclear-staining was performed with Hoechst 33342 (Molecular Probes). Images were obtained using confocal microscopy (Carl Zeiss, LSM700).

### Primary culture of colon mesenchymal cells (CMCs) and macrophages

For isolation of CMCs, colons from 6–10-week-old WT or AKAP12KO mice were dissected, cut into 2-3 mm pieces and washed with HBSS without calcium and magnesium ion (Invitrogen). To remove the epithelial layer, intestinal pieces were then incubated in the HBSS containing 5 mM EDTA and 1 mM DTT for 20 min in shaking incubator at 37 °C two or three times. After being chopped by scissors, the tissue was incubated with 1 mg/ml Collagenase D (Roche), 1 mg/ml Dispase II (Sigma-Aldrich) and 25 U/ml DNase I (Sigma-Aldrich) in DMEM (GenDEPOT) for 60 min in shaking incubator at 37 °C twice. Supernatants were centrifuged and cell pellets were resuspended in DMEM supplemented with 10% FBS (GenDEPOT), 100 U/ml penicillin, 100 μg/ml streptomycin and 0.25 μg/ml amphotericin B and plated in cell culture flasks. The cells at passages 2–3 were used. Bone-marrow derived macrophages (BMDMs) were obtained as described previously^33^. Briefly, femur and tibia were obtained from 8–12-week-old WT or AKAP12KO C57BL/6 mice. After sacrificing a mouse by cervical dislocation, the hind legs of the mouse were dissected and skin and muscle of the leg were removed using sterile scissors and forceps. Under sterile conditions, epiphyses were removed, and the bones were flushed with 20 ml ice-cold sterile PBS by 22-gauge needle syringe through a 70 μm strainer (BD falcon) into 50 ml tube. Filtrates were centrifuged at 500 × *g* and supernatants discarded. Pellets resuspended in RPMI (GenDEPOT), supplemented with 10% FBS, 100 U/ml penicillin, 100 μg/ml streptomycin and 0.25 μg/ml amphotericin B and, plated in cell culture flasks. After 4–6 h incubation at 37 °C, supernatants were collected and resuspended in complete RPMI containing 30% L929 conditioned media. The resupended cell suspension was filtered through a 40 μm strainer plated on petri dishes, and allowed to differentiate at 37 °C for 5 days.

### Collagen gel assay

Collagen type I purified from rat tail (Corning) was used to fabricate three dimensional matrix. Collagen gels were formed by collagen fibril self-assembly inside of 24-well culture plates. Briefly, the pH of the collagen type I solution was adjusted to 7.4 by mixing with 1 M NaOH, NaHCO_3_, H_2_O, 10xRPMI (Sigma Aldrich) and cell suspension. The collagen solutions were incubated at 37 °C for 30 min. After gelation, RPMI was added into collagen gels. After 6 h, gels were released by using fine straight and curved forceps to promote cell based remodeling of matrix. Gels were incubated for 2 days and used for further analysis.

For immunofluorescence, collagen gel matrices remodelled by CMCs were fixed with 4% PFA, dehydrated with sucrose, embedded in OCT compound and sectioned at 10-μm-thickness.

### Flow cytometry

For isolation of colon lamina propria mononuclear cells, procedures were as described for primary culture of CMCs and macrophages until digesting colon lamina propria to dissociate cells from the tissues. Then, the mononuclear cell population was selected from the cell suspension by using Percoll gradient separation. Dissociated cells were resuspended to 40% Percoll solution layered up to 80% Percoll solution. Resuspension were centrifuged at 1000 × *g* at 20 °C without brakes. Carefully collected cells in interphase of layered solutions were washed once with FACS buffer (3% BSA in PBS).

For isolation of cells in collagen gels, gels were digested with digestion solution containing 1 mg/ml Collagenase D (Roche), 1 mg/ml Dispase II (Sigma-Aldrich) and 25 U/ml DNase I (Sigma-aldrich) for 1 hr at 37 °C in a shaking incubator and repeated once. Supernatants were centrifuged at 500 × *g* and washed once with FACS buffer

These suspensions were blocked with mouse CD16/CD32 antibodies for 30 min at 4 °C and stained with V450-conjugated antibodies to mouse CD11b, PE-conjugated antibodies to mouse F4/80, and APC-conjugated antibodies to CD206 (Biolegend) for 1 hr at 4 °C. Then, cells were washed with FACS buffer and analyzed. Samples were acquired with FACSVerse (BD Bioscience) and data were analyzed with FACSuite software.

### Mechanical properties of collagen gels

The mechanical properties of the collagen gels matrix were characterized using Advanced Rheometric Expansion System (ARES). Collagen gels inserted with colon primary mesenchymal cell were remodeled for 2 days and fixed with 4% PFA. The mechanical properties were then measured similar as in a previous report^34^. The storage modulus at 1% strain and at 1 Hz was recorded periodically until the storage modulus reached its equilibrium value (~10 min). Then, a strain sweep was performed to confirm this value was within the linear elastic regime.

### Western blotting

The cells used were homogenized and lysed in buffer [20 mM Tris-HCl (pH 7.5), 150 mM NaCl, 1 mM Na_2_EDTA, 1 mM EGTA, 1% Triton, 2.5 mM sodium pyrophosphate, 1 mM beta-glycerophosphate, 1 mM Na_3_VO_4_, 1 μg/ml leupeptin, and protease inhibitor cocktail. Immunoblotting was performed using primary antibodies against AKAP12 (Santa Cruz, I. Gelman, Roswell Park Cancer Institute) and, α-tubulin (BioGenex).

### Enzyme-linked immunosorbent assay

CMCs and LPS (100 ng/ml) primed BMDMs or LPS-primed BMDMs alone (control) were co-cultured in collagen gels for 2 days and culture supernatant was assayed for TNF-α using mouse TNF-α ELISA MAX standard sets (Biolegend) in coated 96-well plates (Corning). TNF-α concentration was calculated from the standard curve. Results were presented as fold change versus control.

### Bioinformatics

Publicly available datasets of ulcerative colitis patients and DSS induced colitis mouse gene expression microarrays were downloaded from the GEO database (http://www.ncbi.nlm.nih.gov/geo: GSE38713 and GSE22307) and re-analyzed. The expression profiles of AKAP12 and CD206 were extracted from the independent datasets, and quantitative preparation of AKAP12 in each group was performed. The relationship between the expression of AKAP12 and CD206 was determined by correlation analysis. Differentially expressed genes in groups of ulcerative colitis patients and DSS-induced colitis were detected and ranked using GEO2R software in PubMed. Then, genes significantly changed gens (P < 0.001) were functionally categorized according to KEGG pathway using DAVID 6.7 software and the biological processes related with AKAP12 protein function were selected. Significantly correlated genes clusters with AKAP12 (R > 0.8) were also classified with KEGG pathway.

### Data analysis and statistics

Statistical analysis was done with an unpaired two-tailed Student t-test for single comparisons and ANOVA followed by post hoc analysis with Tukey-Kramer test for multiple comparisons.

Results with a P value of <0.05 were considered statistically significant. Data are represented as the means ± S.E.M. Pearson’s coefficients with associated P-values were used for correlation analysis.

## Additional Information

**How to cite this article:** Yang, J.-M. *et al*. Structural environment built by AKAP12+ colon mesenchymal cells drives M2 macrophages during inflammation recovery. *Sci. Rep.* **7**, 42723; doi: 10.1038/srep42723 (2017).

**Publisher's note:** Springer Nature remains neutral with regard to jurisdictional claims in published maps and institutional affiliations.

## Supplementary Material

Supplementary Information
