# Supplementary material for: Structural environment built by AKAP12+ colon mesenchymal cells drives M2 macrophages during inflammation recovery
Source: Sci Rep. 2017 Feb 16;7:42723. doi: 10.1038/srep42723 (PMC5311874; doi:10.1038/srep42723)
Supplement: Supplementary Information [file srep42723-s1.pdf]

Supplementary Information

Structural environment built by colon mesenchymal cells drives M2 macrophages during inflammation recovery

Jun-Mo Yang[1], Hye Shin Lee[1], Ji Hae Seo[1], Ji-Hyeon Park[1], Irwin H. Gelman[2], Eng H. Lo[3] and Kyu-Won Kim[1, 4*]

[1]SNU-Harvard NeuroVascular Protection Research Center, College of Pharmacy and Research Institute of Pharmaceutical Sciences, Seoul National University, Seoul ,151-742, Korea.

[2]Department of Cancer Genetics, Roswell Park Cancer Institute, Buffalo, NY 14263, USA

3Neuroprotection Research Laboratory, Departments of Radiology and Neurology, Massachusetts General Hospital and Harvard Medical School, Charlestown, MA 02129, USA

[4]Crop Biotechnology Institute, GreenBio Science and Technology, Seoul National University Pyeongchang 25354, Republic of Korea

Supplementary Figure 1

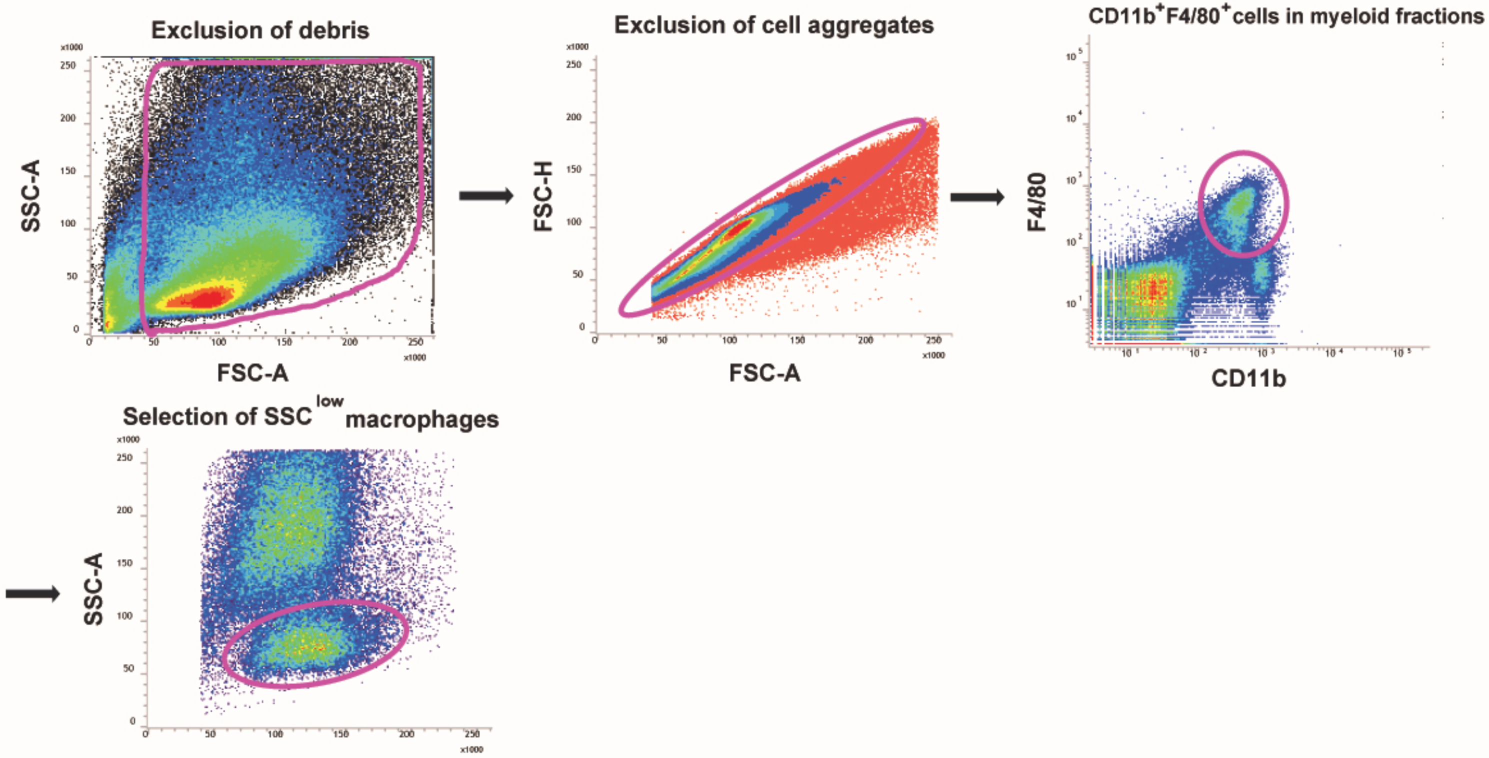

Supplementary Figure 1. Gating strategy for isolating the macrophage population in DSS-induced mouse colons Cells in pink circle are gated. First, whole prepared cell populations were gated. Then, singlets of the cells were gated. CD11b+ F4/80+ cell included

Supplementary Figure 2

A

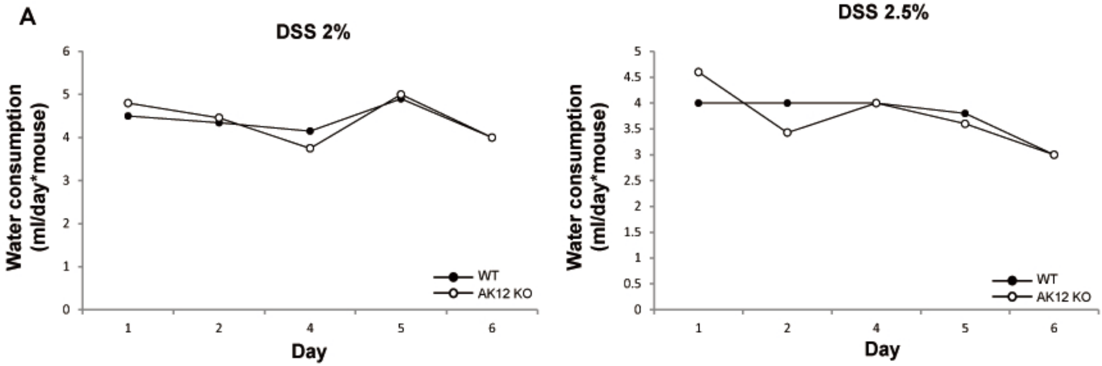

B

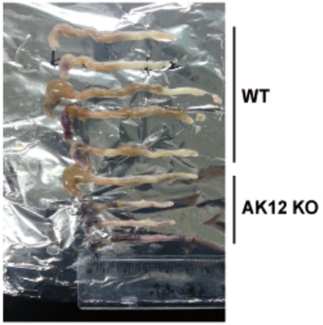

Supplementary Figure 2. DSS-induced colitis model (A) Consumption of DSS in water per cage was calculated from diminished volume of water and then divided by number of mouse. **(B)** Picture of DSS-induced colon of WT an AKAP12 KO mice with ruler was taken at 12 day

Supplementary Figure 3

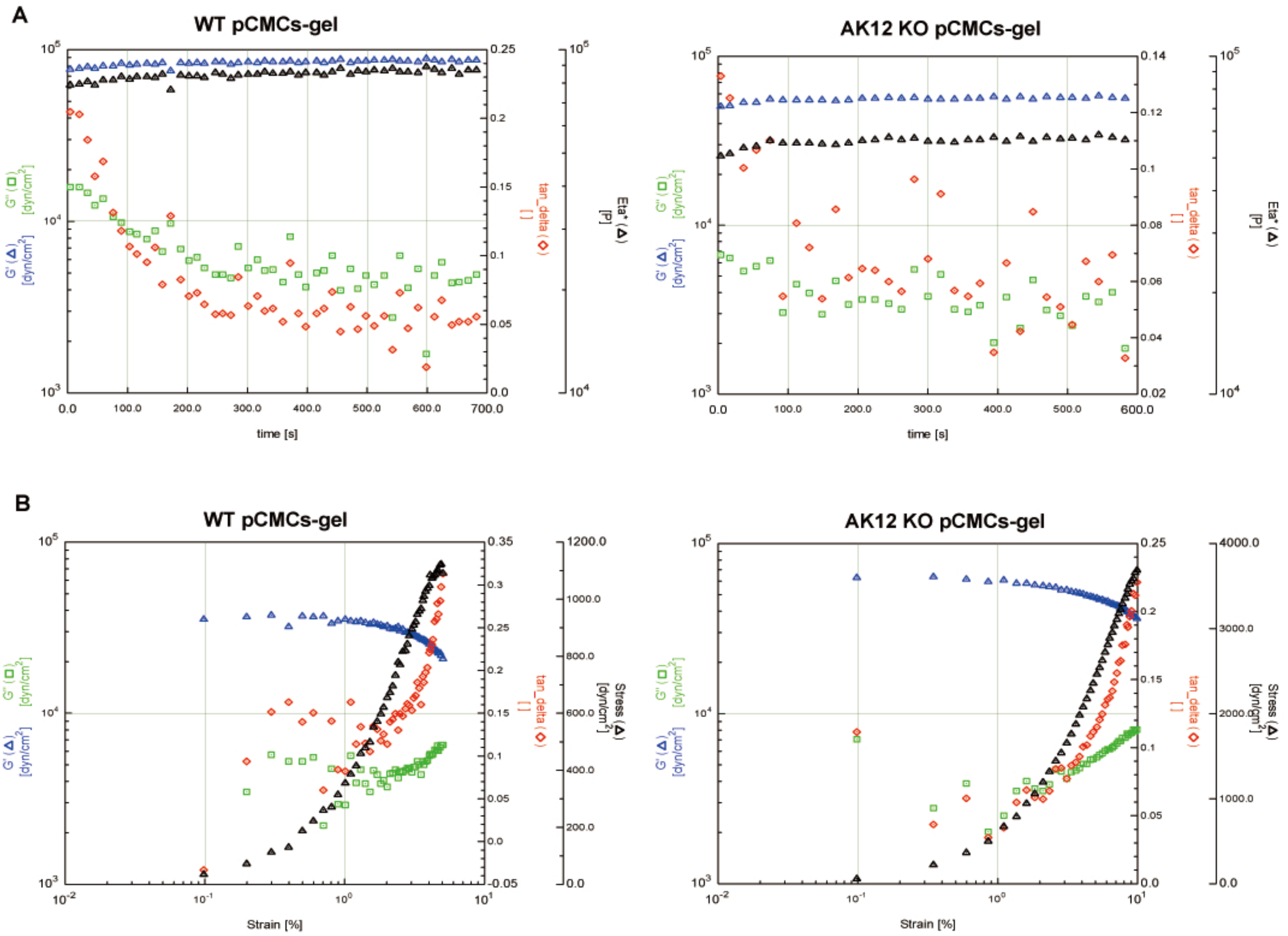

Supplementary Figure 3. Measurement of the stiffness of remodelled collagen gels (A) Representative chart of periodically modulus in WT and AKAP12 KO pCMCs mediated collagen gels. **(B)** Representative chart of recorded modulus versus strain in WT and AKAP12 KO pCMCs-gels.

Supplementary Figure 4

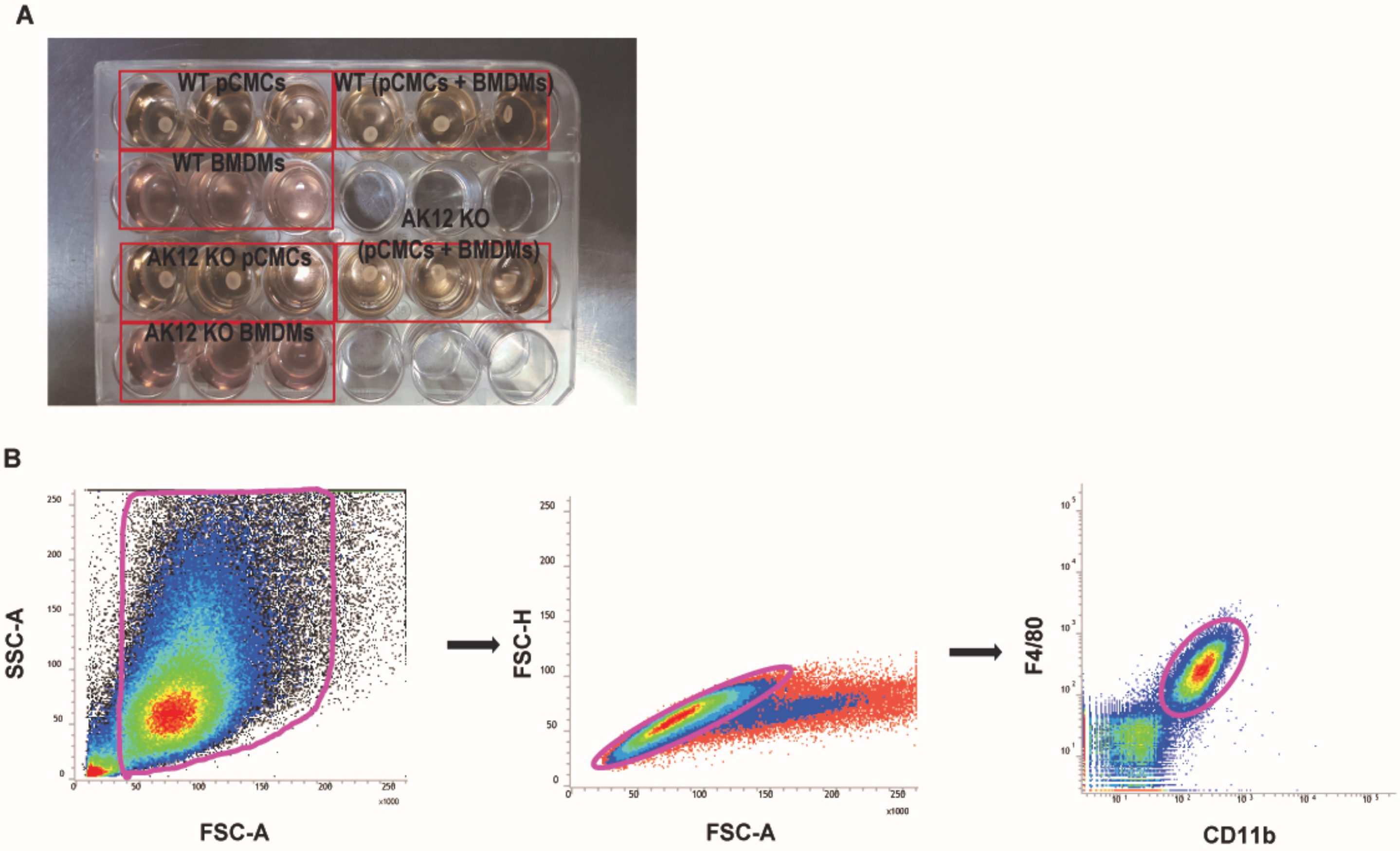

Supplementary Figure 4. CMCs mediated collagen gel remodeling (A) Pictures of WT and AKAP12 KO pCMCs, BMDMs and pCMCs+BMDMs gels after incubating 48 hr. **(B)** Gating strategy for isolating inserted BMDMs in gels. Cells in pink circle were gated.

Supplementary Table

DSS concentration (%)	Day	N	More severe damaged	Statistic significance (P)
3	10	5	AKAP12KO	0.0432
2	9	10	AKAP12KO	0.0058
2.5	10	5	AKAP12KO	0.0198
2	8	10	AKAP12KO	0.0063
2.5	(-)	10	(-)	NS
2	(-)	5	(-)	NS
2	8	10	AKAP12KO	0.0054
2	(-)	8	(-)	NS
2	9	6	AKAP12KO	0.0013
2	8	7	AKAP12KO	7.14036E-06

Supplementary Table. The accumulated results of DSS-induced colitis experiment In almost of the experiment**,** AKAP12 KO mice were more damaged during intestinal inflammation. DSS concentration, day at measured, number of mouse and statistical significance were recorded.
